# Supplementary material for: TLR-exosomes exhibit distinct kinetics and effector function
Source: Sci Rep. 2017 Mar 14;7:41623. doi: 10.1038/srep41623 (PMC5349571; doi:10.1038/srep41623)
Supplement: Supplementary Information [file srep41623-s1.pdf]

Supplementary: TLR-exosomes exhibit distinct kinetics and effector function

Swetha Srinivasan, Michelle Su, Shashidhar Ravishankar, James Moore, PamelaSara E Head, J Brandon Dixon, Fredrik O Vannberg

Supplementary figure 1: Characterization of exosomes used in the study

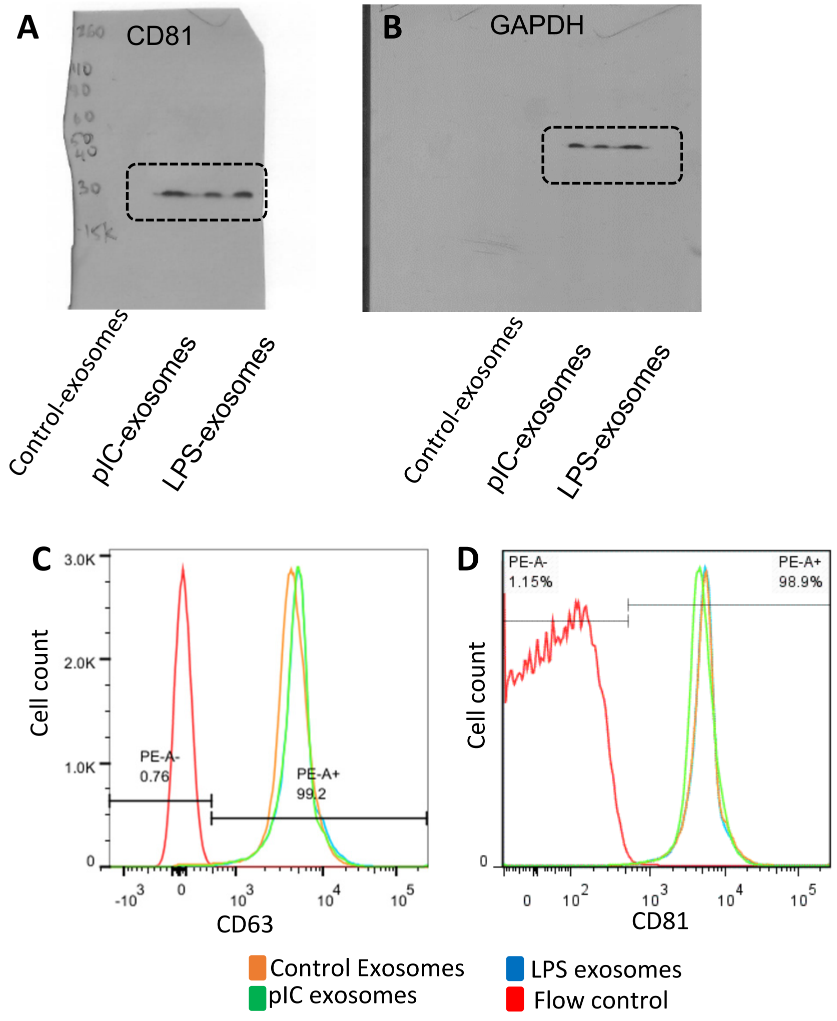

Supplementary figure 1: Characterization of exosomes used in the study. (**a**) Complete western blot of CD81 with control, pIC and LPS exosomes. Flow cytometry showing (**b**) CD63 and (**c**) CD81 levels on the exosomes.

Supplementary figure 2: Estimating TLR agonist carryover by exosomes

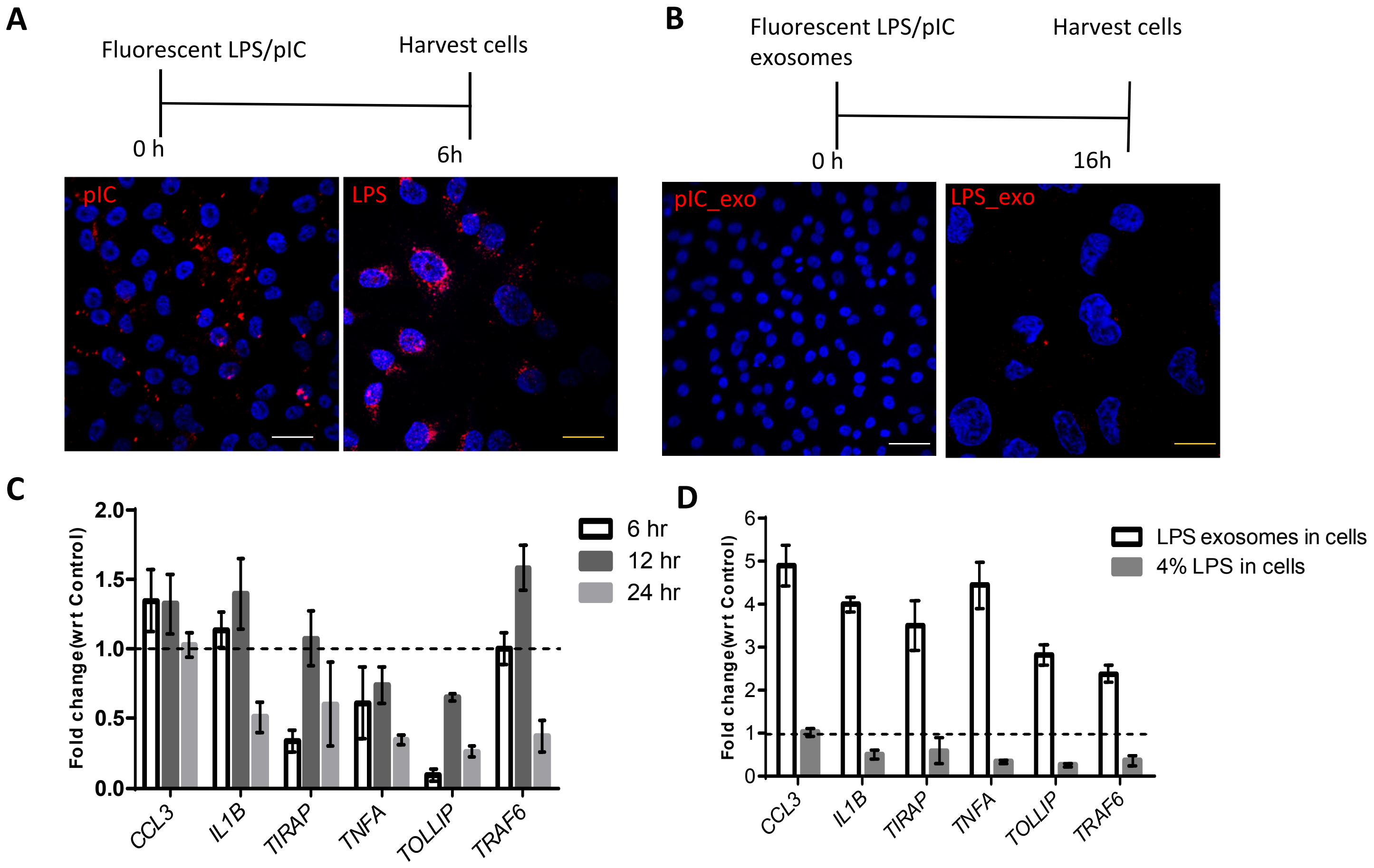

Supplementary figure 2:. Estimating the carryover of TLR agonist from local cells to distal cells by exosomes. Confocal images showing (**a**) LPS-AF594 and pIC- Rhodamine uptake by parental cells and (**b**) exosomes from local cells treated with LPS-AF594 and pIC- Rhodamine added to distal cells to show no PIC and 4% LPS carryover. Scale bars, 50 μm. (**c**) Time course of gene expression in local cells after stimulation with 4% LPS and (**d**) Comparison of gene expression at 24 hours between local cells stimulated with 4% LPS and distal cells stimulated with LPS exosomes.

Supplementary Fig. 3: The LPS response in parent cells and recipient cells

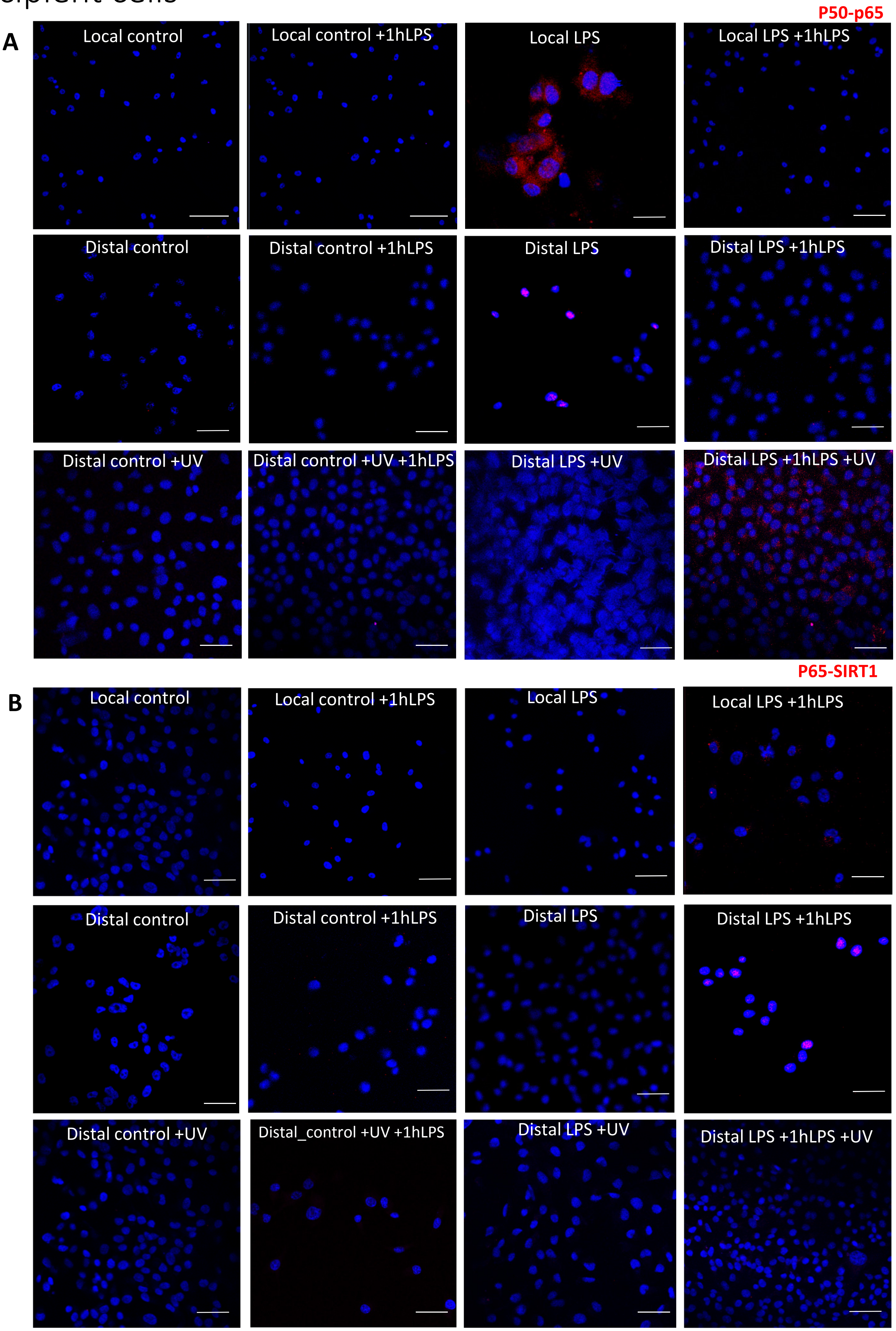

Supplementary Fig. 3: The LPS response in local and distal cells. Proximity ligation assay showing (**a**) the P50-P65 co-localization and (**b**) P65-SIRT1 in the cells indicated

Supplementary Figure 4: Pathways analysis of distal cells from microarray data

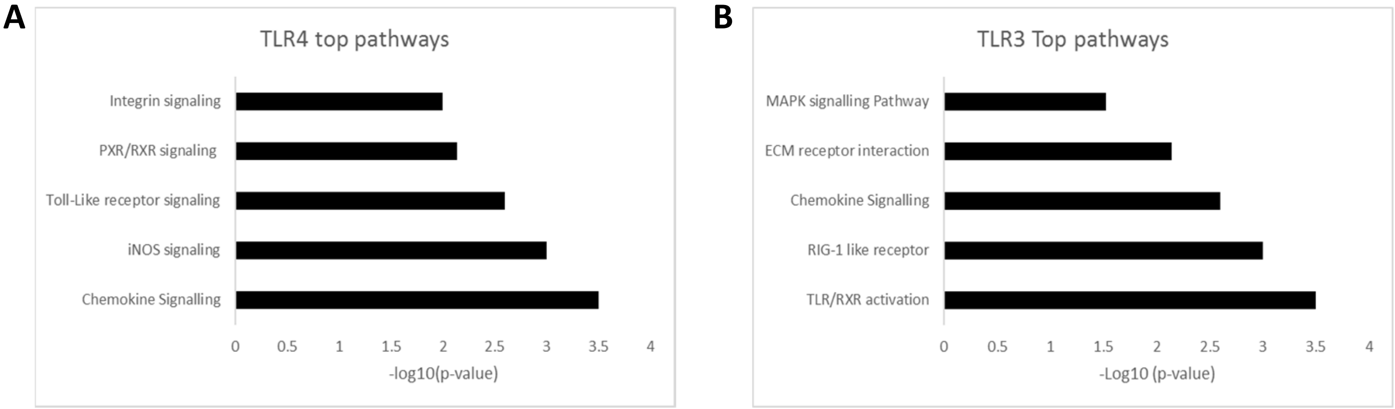

Supplementary Figure 4: Pathways analysis of distal cells from microarray data. Pathways enriched in distal cells stimulated with (**a**) LPS exosomes and (**b**) pIC exosomes

Supplementary Fig. 5: Effect of UV on the nucleic acid content of exosomes

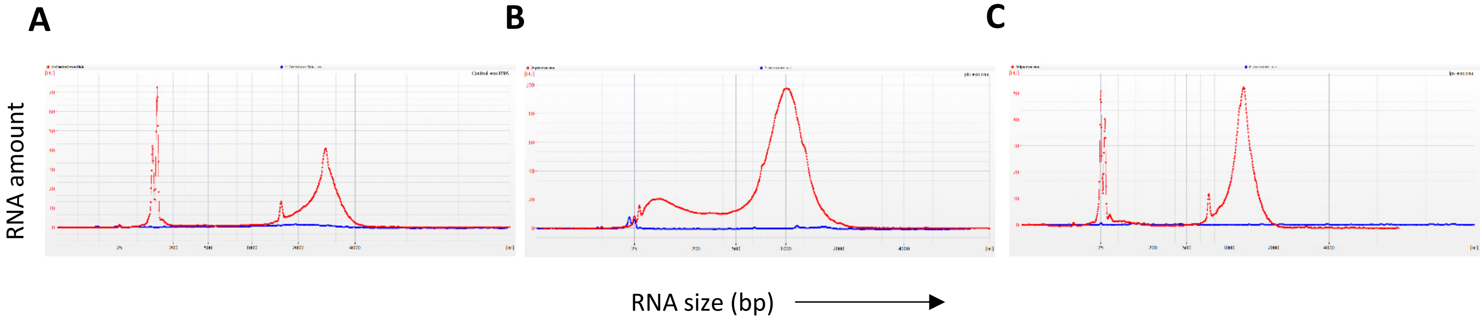

Supplementary Fig. 5: Effect of UV on nucleic acid content of exosomes. RNA size distribution profiles obtained on a Bioanalyzer pico RNA chip of (**a**) Control exosomes , (**b**) pIC exosomes, and (**c**) LPS exosomes ; before (red lines) and after UV treatment (blue Line)

Supplementary Fig. 6: RNA-Seq of distal macrophages

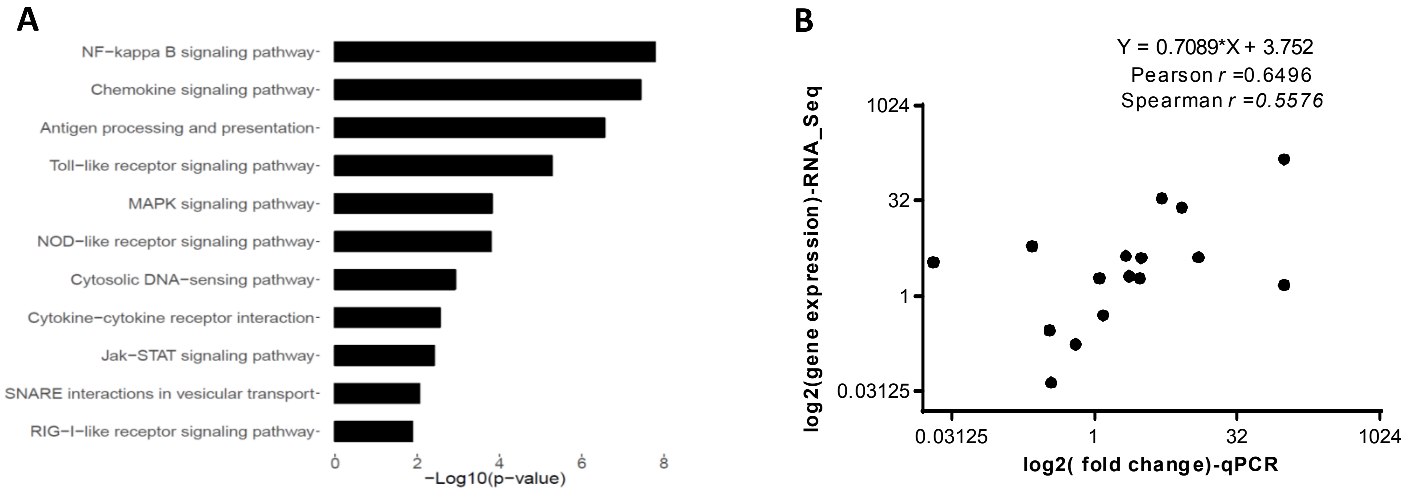

Supplementary Fig. 6: RNA-Seq of distal macrophages. (a) Pathways enriched in distal macropages with pIC exosomes as compared to PBS. (**b**) Scatter plots showing the correlation between the fold change detected via qCR when compared to RNA-Seq

Supplementary Fig. 7: Macrophages retain both control and pIC exosomes

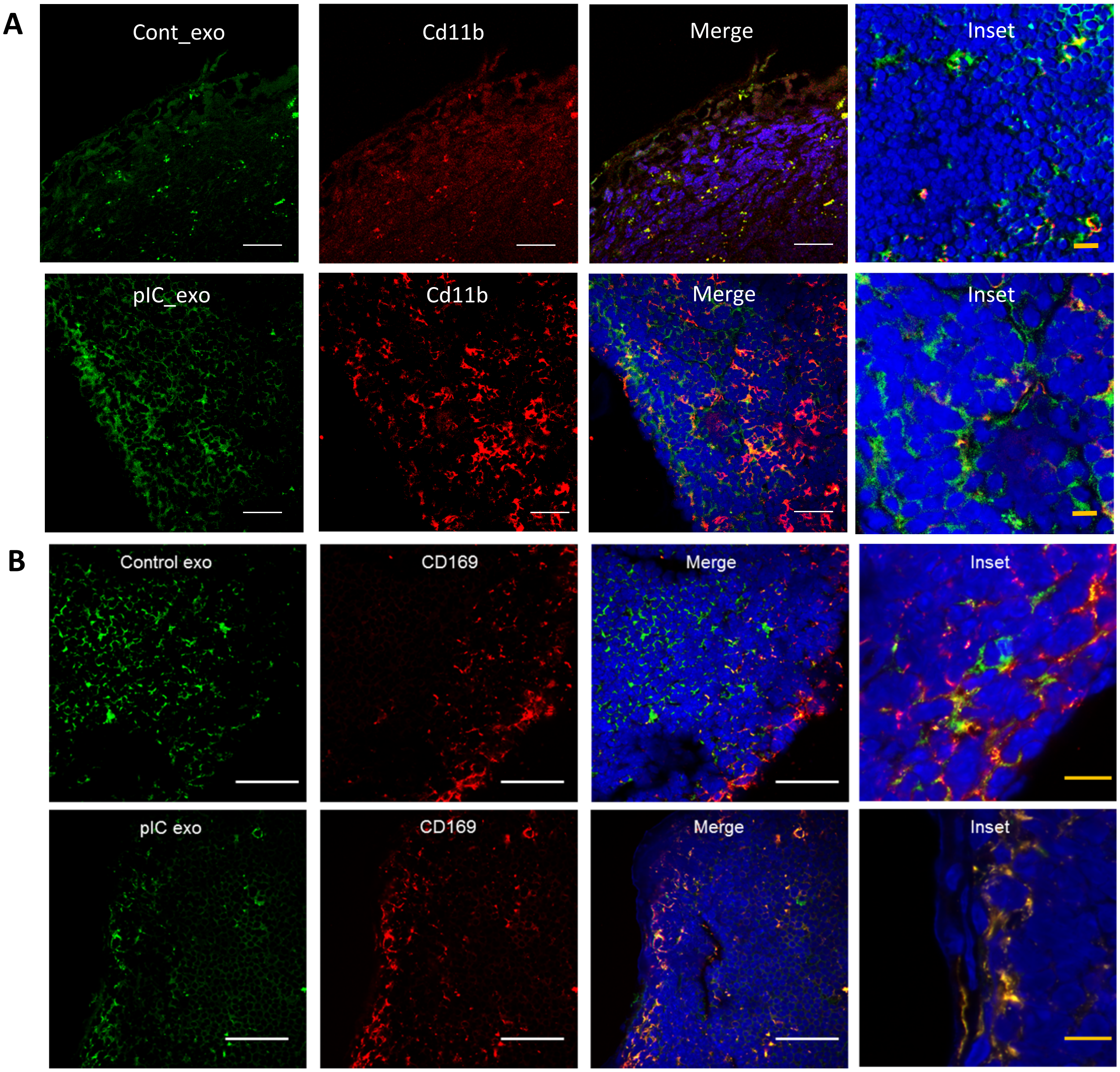

Supplementary Fig. 7: Macrophages retain both control and pIC exosomes **(a)** CD11b+ macrophages and (b) CD169+ subcapsular sinus macrophages retain control and pIC-exosomes.

Supplementary Fig. 8: Impact of control exosomes in whole node sections

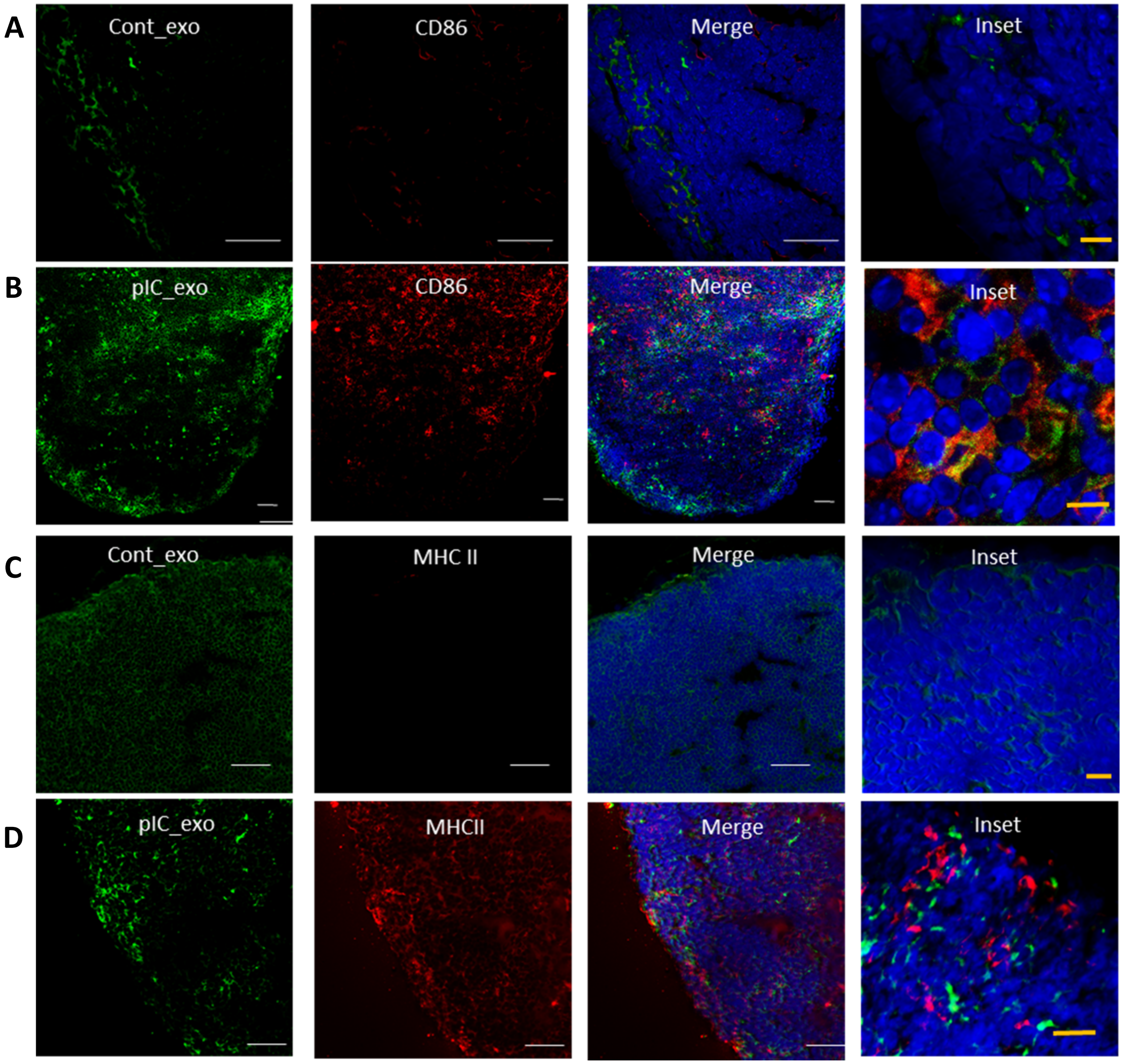

Supplementary Fig. 8: Validation of M1 markers Cd86 and MhcII expression in lymph node sections after exposure to control or pIC exosomes. White scale bars, 50 μm, yellow scale bars, 10 μm.

Supplementary Fig. 9: Lymphatic transport and retention of exosomes

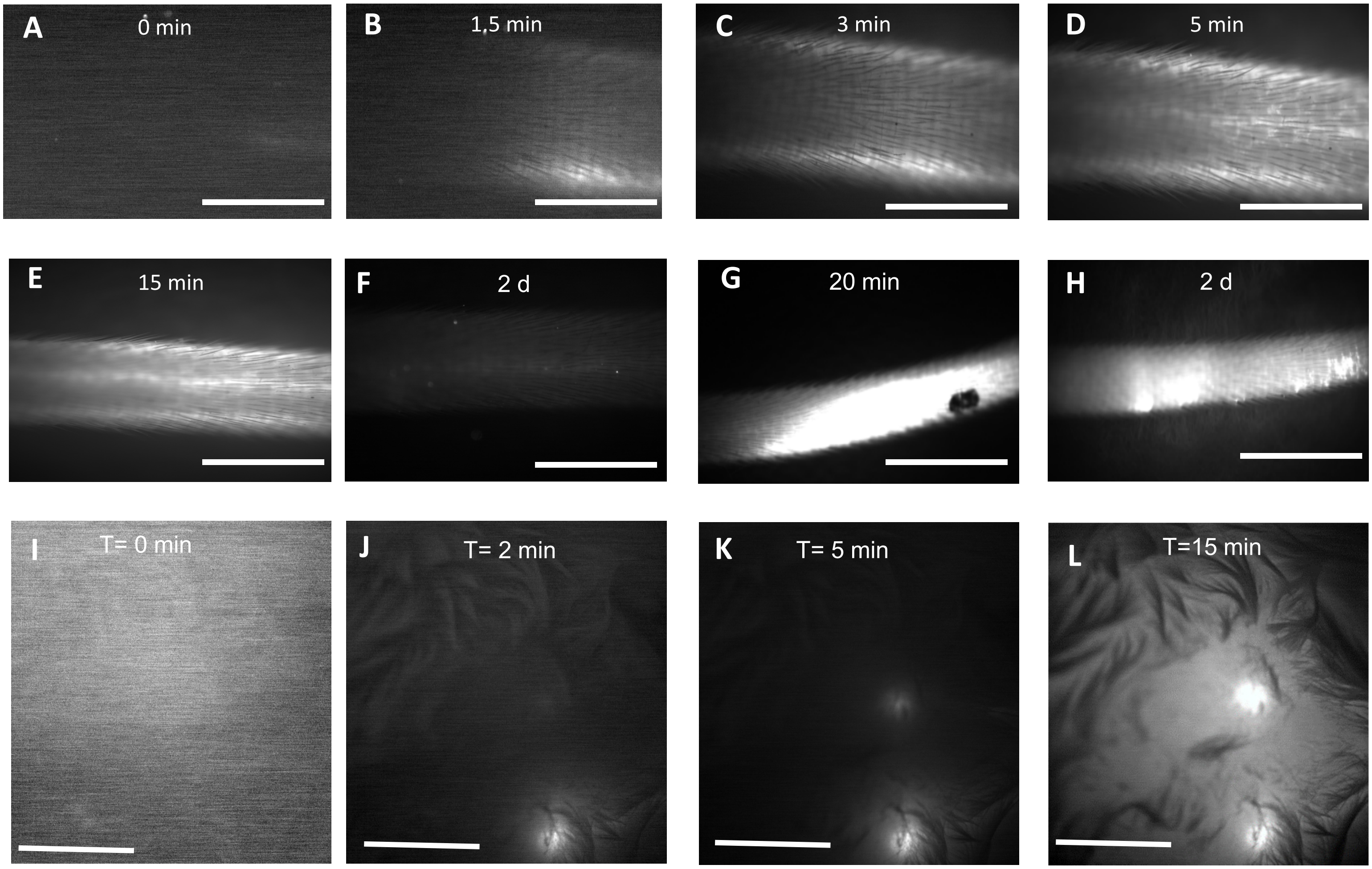

Supplementary Fig. 9: Lymphatic transport and retention of exosomes. pIC exosomes are seen in the lymphatic collecting vessels at (**a**) 0 mins, (**b**) 1.5 mins, (**c**) 3 mins, (**d**) 5mins , (**e**) 15 mins and (**f**) 2 days. The injection site is shown at (**g**) 20 mins and (**h**) 2 days. Exosomes are detected in the draining lymph node at (**i**) 0 mins, (**j**) 2mins, (**k**) 5 mins and (**l**) 15 mins

Supplementary Fig. 10: Kinetics of exosome transport in the lymphatics

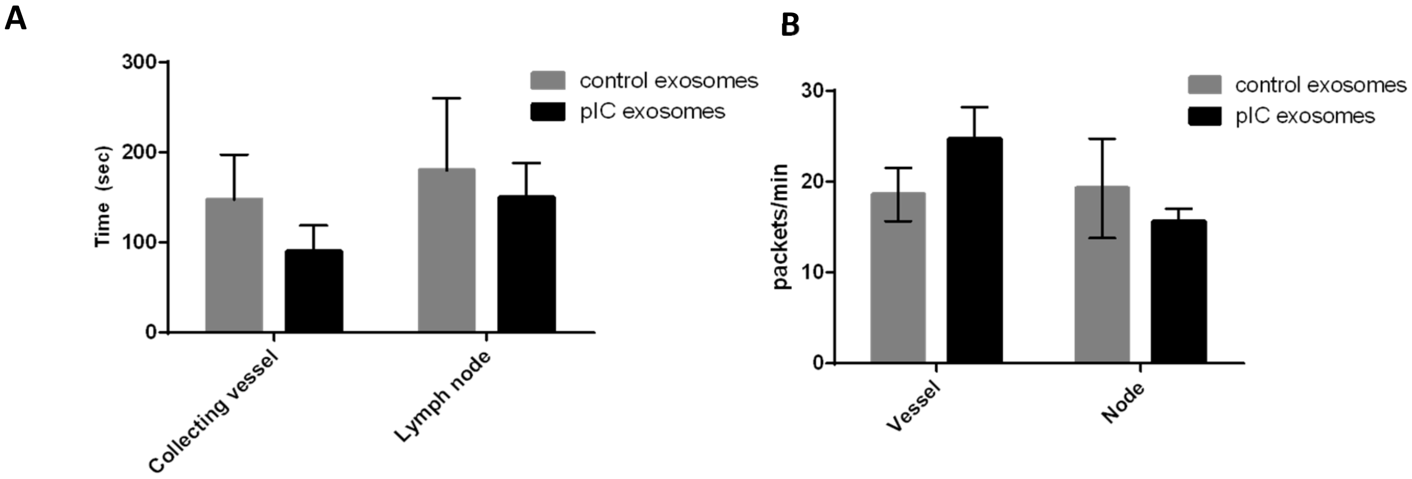

Supplementary Fig. 10: Kinetics of exosome transport in the lymphatics. (**a)** Arrival time of detectable levels of fluorescence for collecting vessels and draining lymph nodes. (**b**) Packet frequency of control and pIC exosomes in the collecting lymphatic vessels and nodes.

Supplementary Fig. 11: Comparison of gene expression changes in macrophages with free_pIC

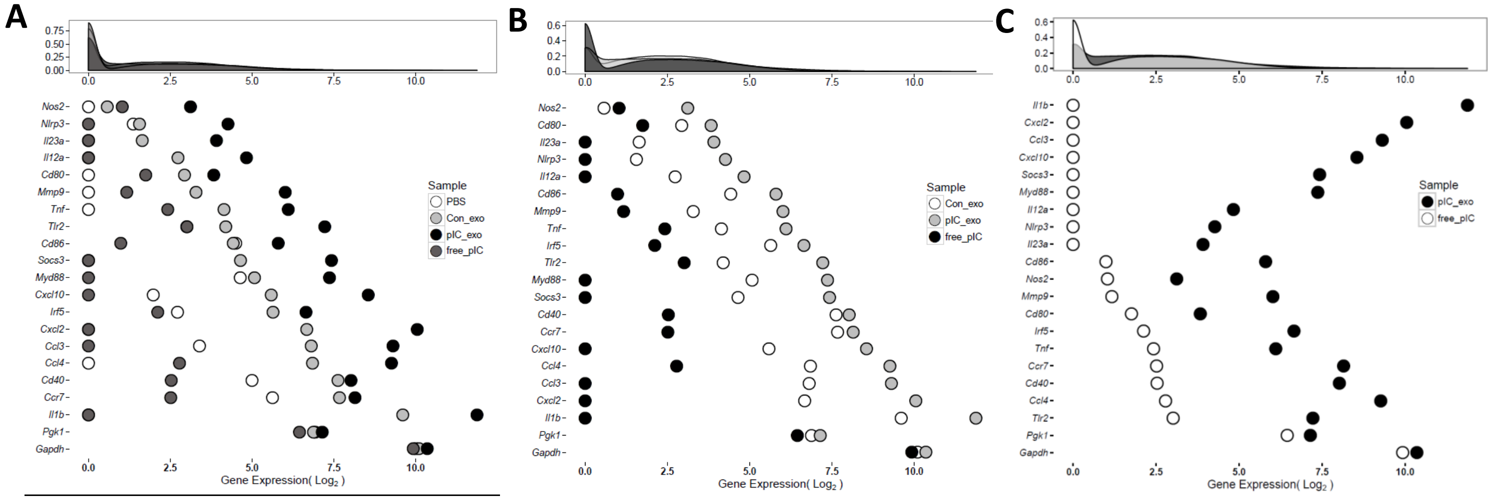

Supplementary Fig. 11: Comparison of gene expression changes in macrophages with free_pIC.. Relative expression of key macrophage markers from lymph nodes after exposure to (**a**) PBS, control exosomes, pIC exosomes or free pIC (**b**) control exosomes, pIC exosomes or free pIC and (**c**) pIC exosomes or free pIC

Supplementary Fig. 12: Comparison of gene expression changes in whole lymph nodes with free_pIC

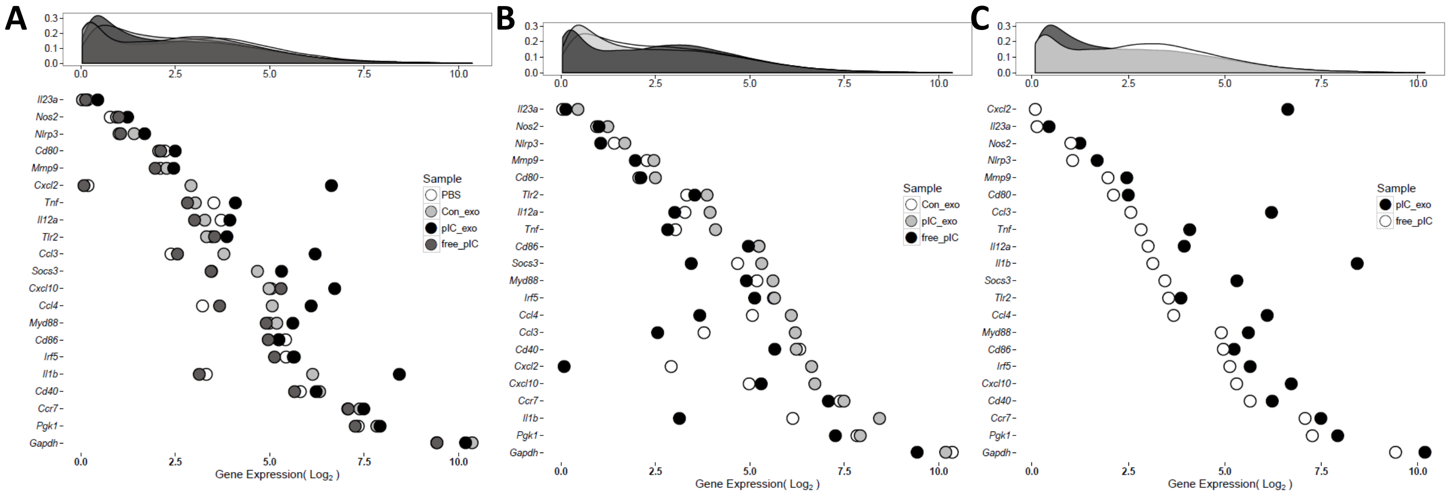

Supplementary Fig. 12: Comparison of gene expression changes in whole lymph nodes with free_pIC. Relative expression of key neutrophil markers in whole lymph nodes after exposure to (**a**) PBS, control exosomes, pIC exosomes or free pIC (**b**) control exosomes, pIC exosomes or free pIC and (**c**) pIC exosomes or free pIC

Supplementary Videos

Supplementary Video 1: Example video of pIC exosome arrival in the collecting vessels of a mouse 10 cm downstream from the site of intradermal injection. The dominant vessel is seen below and the nondominant vessel is seen above. Video is played at 10X speed

S**upplemental Video 2:** Example video of pIC exosome arrival in the draining (sciatic) lymph nodes of a mouse within minutes of intradermal exosome injection at the tip of the tail. The dominant node is seen below and the non-dominant vessel is seen above. Video is played at 10X speed
